# Supplementary material for: Brimonidine and timolol concentrations in the human vitreous and aqueous humors after topical instillation of a 0.1% brimonidine tartrate and 0.5% timolol fixed-combination ophthalmic solution: An interventional study
Source: PLoS One. 2022 Dec 1;17(12):e0277313. doi: 10.1371/journal.pone.0277313 (PMC9714730; doi:10.1371/journal.pone.0277313)
Supplement: S1 File — (PDF) [file pone.0277313.s003.pdf]

Machine Translated by Google

# Translocation of brimonidine to the human vitreous in ibeta ophthalmic solution

# research on

[Medical institution]

Name of medical institution: University of Fukui

Hospital Principal investigator: Professor Dai Inatani,

Ophthalmologist Funding: Senju Pharmaceutical Co., Ltd.

**Version: 1.1**

**Created: March 2, 2020**

Machine Translated by Google

table of contents


1 research summary ................................................ Four
1.1 Overview ................................................ Four
1.2 Schematic ................................................ 6
1.3 Research schedule ................................................ 7
2 Background ................................................ 7
2.1 background ................................................ 7
2.2 Significance of research ................................................ 8
3 Objectives and evaluation items ................................................ 9
Four Study design ................................................ 9
4.1 Study design ................................................ 9
4.2 Randomization/blinding ................................................ 9
Five Target population ................................................ 9
5.1 Eligibility Criteria ................................................ 9
5.1.1 Selection criteria ................................................ 9
5.1.2 Exclusion Criteria ................................................ Ten
5.1.3 Basis for setting ................................................ Ten
5.2 Target sample size ................................................ Ten
5.2.1 Target sample size ................................................ Ten
5.2.2 Basis for setting the number of cases ................................................ Ten
6 Intervention ................................................ Ten
6.1 Medicines and medical devices used ................................................ Ten
6.1.1 Overview of pharmaceuticals and medical devices ................................................ 11
6.1.2 Anticipated side effects ................................................ 11
6.2 Protocol treatment ................................................ 12
6.2.1 Dosage/Dosage ................................................ 12
6.2.2 Packaging/Labeling ................................................ 13
6.3 Management method ................................................ 13
6.3.1 Storage/delivery ................................................ 13
6.3.2 Disposal/Return ................................................ 13
6.4 Combination therapy ................................................ 13
6.4.1 Concomitant treatment ................................................ 13
6.4.2 Concomitant therapy ................................................ 14
6.4.3 Concomitant restrictive therapy ................................................ 14
6.4.4 Precautions for concomitant use ................................................ 14
7 Research methods and procedures ................................................ 15
7.1 Subject Recruitment ................................................ 15
7.2 Obtaining Consent ................................................ 15

Machine Translated by Google

7.3 Subject Enrollment ................................................ ................................................ ................................................15

7.4 Observation items and information/procedures to be collected ................................................ ................................................ .......16

7.5 Implementation period and registration period.......................................... ................................................ ......................17

7.6 End of Registration Period and Observation Period.......................................... ................................................ .............17

8 How to obtain consent ................................................................ ................................................ ................................................18

8.1 Informed Consent ................................................................. ................................................ ...........18

8.2 Withdrawal of Consent................................................ ................................................ ................................................18

9 Adverse Events and Illnesses, etc.......................................... ................................................ ................................................18

9.1 Definitions................................................ ................................................ ................................................ .18

9.1.1 Adverse event definition ................................................ ................................................ .............19

9.1.2 Definition of Serious Adverse Events ................................................ ................................................ .....19

9.1.3 Definition of diseases, etc. ................................................ ................................................ ................................19

9.2 Research and causality.......................................... ................................................ ................................19

9.3 Predictability ................................................ ................................................ ................................................20

9.4 Collection period and follow-up period.......................................... ................................................ ......................20

9.5 Adverse event reporting ................................................. ................................................ ................................20

9.5.1 Reporting of all adverse events ......................................................... ................................................ ....20

9.5.2 Reporting of Serious Adverse Events ................................................ ................................................ .....20

9.5.3 Illness Report ................................................................. ................................................ .................20

Ten Cancellation and Termination........................................ ................................................ ................................................ ..twenty one

10.1 Subject discontinued......................................................... ................................................ ..............................twenty one

10.2 Discontinuation of study.......................................... ................................................ ........................................twenty two

10.3 End of research................................................ ................................................ ...........................................twenty three

11 Methods of Minimizing Anticipated Benefits/Disadvantages and Risks ................................................ .....................................twenty three

11.1 Projected profit ................................................................ ................................................ .................................twenty three

11.2 Anticipated Disadvantages.......................................... ................................................ ..............................twenty three

11.3 How to minimize risk......................................................... ................................................ ....................twenty three

12 Ethical and Sensitive Considerations.......................................... ................................................ ............................twenty four

12.1 Observance of Laws and Guidelines........................................ ................................................ ...............................twenty four

12.2 Accredited Clinical Research Review Board ................................................ ................................................ ....................twenty four

12.3 Handling of personal information, etc.......................................... ................................................ .......................twenty four

12.3.1 About anonymization ................................................................ ................................................ .............twenty four

12.3.2 Rights of Subjects, etc. Regarding Personal Information.......................................... ...................................twenty five

12.4 Compensation for health damage ................................................................................ ................................................ ......................twenty five

12.5 Subject's financial burden or honorarium ........................................ ................................................ .............twenty five

13 Research Funding, Conflicts of Interest and Disclosure of Information.......................................... ................................................ ..............twenty five

13.1 Sources of research funding.......................................... ................................................ .....................................twenty five

13.2 Conflict of Interest Situation........................................ ................................................ ................................26

Machine Translated by Google


13.3 Method of Information Disclosure.......................................... ................................................. .................................26

13.4 Publication of results .............................................................. ................................................. ................................................26

14 Statistical Matters................................................. ................................................. ................................................26

14.1 Analysis population ................................................. ................................................. ................................................26

14.2 Statistical analysis............................................... ................................................. ................................................27

14.2.1 Analysis of the primary endpoint ................................................................. ................................................. .......27

14.2.2 Analysis of secondary endpoints ................................................................. ................................................. ........27

14.3 Statistical Analysis Plan Changes....................................... ................................................. ................................28

15 Creating a case report form................................................. ................................................. ................................................28

16 Storage and Disposal of Specimens and Information.......................................... ................................................. ................................28

16.1 Storage method/storage period.......................................... ................................................. ...............................28

16.1.1 Sample storage method/storage period ............................................ ................................................28

16.1.2 Storage method and storage period of information ................................................ ................................................28

16.2 Disposal method ............................................... ................................................. ...............................................29

16.2.1 How to dispose of samples ........................................... ................................................. .............29

16.2.2 How we dispose of information ............................................... ................................................ .............29

16.3 Safety management method ................................................................ ................................................. ................................................29

17 Quality Control and Quality Assurance.......................................... ................................................. ................................29

17.1 Source material.......................................... ................................................. ................................................29

17.2 Data management ................................................................ ................................................. ................................30

17.3 monitoring................................................ ................................................. ................................................30

17.4 audit................................................. ................................................. ................................................ .30

18 Legal reporting and information sharing, etc................................................. ................................................. .................30

18.1 Who to report to and scope of information sharing........................................ ................................................. .................30

18.2 Creation/change of implementation plan, etc.......................................... ................................................. ................................31

18.3 Periodic reporting ................................................ ................................................. ................................................31

18.4 Non-conformance report ................................................. ................................................. ...............................................31

18.5 Illness Report........................................... ................................................. ................................................32

18.6 Report on study discontinuation................................................. ................................................. ...................32

18.7 Report on completion of research and preparation of report ................................................ ................................................32

18.8 others................................................ ................................................. ................................................33

19 Research system ................................................ ................................................. ................................................. .....33

19.1 Research organization ................................................ ................................................. ...............................................33

19.2 Consultation counter....................................... ................................................. ................................................34

19.3 Subcontracting................................................ ................................................. ................................................34

20 others................................................ ................................................. ................................................. ........34

20.1 Revision history................................................ ................................................. ................................................34

twenty one References................................................................ ................................................. ................................................. .....35

Machine Translated by Google



# 1 Research overview

## 1.1 Overview

| | |
|---|---|
| Title of research: Research on translocation of brimonidine to the human vitreous in ibeta ophthalmic solution | |
| Purpose of research | The only definitive evidence-based treatment for glaucoma is lowering intraocular pressure. However, there have been reports of cases in which visual field loss progresses even when the intraocular pressure is sufficiently lowered. Therefore, attention is focused on new treatments such as neuroprotection that do not rely on lowering intraocular pressure.<br>It is Brimonidine, which is contained in ibeta ophthalmic solution, has an intraocular pressure-lowering effect.<br>In addition to being useful, it is known to have neuroprotective effects. Therefore, in this study, we investigated the possibility of drug therapy for retinal neuroprotection with ibeta ophthalmic solution.<br>Translocation of brimonidine to the vitreous body when ibeta combination ophthalmic solution was administered to patients<br>Evaluate gender. |
| Purpose | Translocation of brimonidine to the vitreous body following ocular administration of ibeta ophthalmic solution to humans<br>Evaluate behavior. |
| Evaluation item | Primary endpoint:<br>[Pharmacokinetic analysis]<br>Brimonidine concentration in aqueous humor and vitreous<br>Secondary endpoints:<br>[Pharmacokinetic analysis]<br>Aqueous humor and vitreous timolol concentrations Correlation<br>between drug concentration data and patient background<br>[Safety evaluation]<br>Adverse events, visual acuity, intraocular pressure, ophthalmologic findings (cornea, conjunctiva), blood pressure, pulse, |
| research design | Single-center, unshielded, uncontrolled study |
| subject | subject<br>Patients with premacular membrane or macular hole who underwent vitrectomy<br>Basis for setting<br>In this study, the vitrectomy was performed to examine the transfer of brimonidine to the vitreous.<br>Aimed at people with plans<br><br>Selection criteria:<br>1) Subject's voluntary written consent after receiving sufficient explanation for participation<br>who can get |

Machine Translated by Google



| | |
|---|---|
| | 2) Aged 20 years or older at the time of informed consent, regardless of gender 3) Patients scheduled for surgery for premacular membrane or macular hole<br><br>Exclusion Criteria:<br>1) Subjects with eye inflammation<br>2) Vitreous hemorrhage<br>3 ) Proliferative diabetic retinopathy 4) Severe corneal epithelial disorder<br>Those who have been judged inappropriate for participation in this study by the appointed physician, etc.<br>6) Subjects with a history of serious adverse reactions to ÿ2 agonists and ÿ- blockers |
| Eyes to be evaluated Eyes undergoing vitrectomy | |
| intervention | Test drug: ibeta combination ophthalmic solution<br><br>Instill 1 drop of the study drug into the eye to be evaluated, twice a day for 8 days (7 days before surgery and on the morning of surgery and 2 hours before surgery (± 1 hour)). |
| Target sample size 10 | |
| Research period | Study period: jRCT publication date to December 31, 2020 Registration period: jRCT publication date to September 25, 2020 Observation period: jRCT publication date to September 30, 2020 Participation period Observation period: 7 days before and after |
| surgery 8 days in total | |
| Concomitant Contraindications There are no concomitant therapies in this study. | |
| research organization | Implementing medical institution/Investigator University of Fukui Hospital Department of Ophthalmology Dai Inatani Professor |

Machine Translated by Google



1.2 Schematic diagram

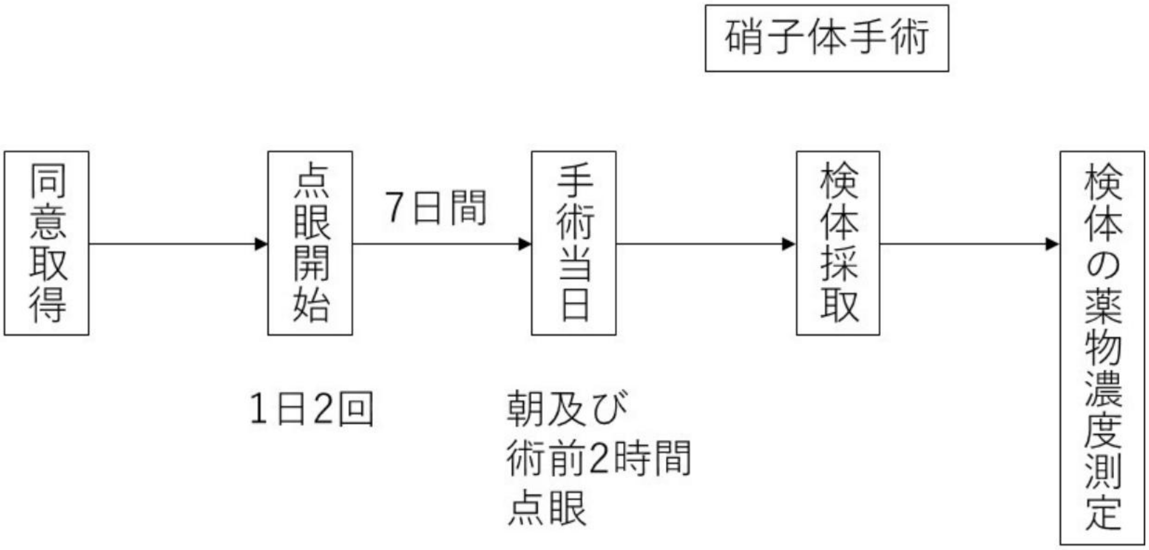

Machine Translated by Google



## 1.3 Research schedule

| | Visit 1 | | Visit 2 | Visit 3 | | | |
|---|---|---|---|---|---|---|---|
| | | Day 1 of administration ~ Day 6 | Day 7 of administration | Day 8 of administration (±1 day) | | | cancel drop out Time |
| | | 7 days before surgery ~2 days ago | day before surgery | On the day of surgery | | | |
| | | | | Morning | 2 hours before surgery | During surgery | |
| Consent acquisition | X | | | | | | |
| Background factors | X | | | | | | |
| Eye drop a | | X | X | X | Xb | | |
| height, weight· BMI | X | | | | | | |
| tear volume | X | | | | | | |
| Axial length | Xc | | | | | | |
| corneal thickness | Xc | | | | | | |
| Corneal curvature radius | Xc | | | | | | |
| Number of corneal endothelial cells | Xc | | | | | | |
| eyesight | X | | X | | | | X |
| intraocular pressure | X | | X | | | | X |
| ophthalmic findings | X | | Xd | | | | Xd |
| Blood pressure/pulse | X | | X | | | | X |
| Specimen collection | | | | | | X | |
| Instillation status | | | X | | X | | X |
| Confirmation of adverse events | | X | | | | | X |

a: If you cannot come to the hospital 7 days before surgery due to a holiday, etc., allow 1 day before and after the period of instillation.

b: The allowable time for instillation 2 hours before surgery is ±1 hour.

c: If conducted within the past 120 days from Visit 1, it can be used as data for Visit 1.

d: Ophthalmic findings should be limited to the cornea and conjunctiva.

# 2 Background

## 2.1 Background

Glaucoma presents with characteristic changes in the optic nerve and visual field, usually by lowering intraocular pressure sufficiently to cause optic nerve damage. It is a disease characterized by functional and structural abnormalities of the eye that can ameliorate or suppress disability1 ). blindness worldwide Approximately 5% of Japanese over the age of 40 develop it2 ), and it is the leading cause of blindness. 3 ). The impairment of visual function due to glaucoma is irreversible and greatly affects the quality of life (QOL) of patients. Therefore, early detection and early treatment are required.

Currently, the only evidence-based definitive treatment for glaucoma is lowering intraocular pressure1 ). However, there are cases in which symptoms characteristic of glaucoma appear despite normal intraocular pressure.

Machine Translated by Google



called pressure glaucoma. Epidemiological studies have revealed that normal tension glaucoma accounts for 72.0% of glaucoma cases in Japan2 ). It has been shown that visual field loss progresses in some patients with normal-tension glaucoma even after a 30% reduction in intraocular pressure, which is considered useful for visual field preservation 4) 5) . For these reasons, the development of new neuroprotective treatments that do not rely on lowering intraocular pressure is attracting attention.

Brimonidine is an intraocular pressure-lowering drug developed by Allergan in the United States. Selectively activating ÿ2 receptors It exerts an intraocular pressure-lowering effect by suppressing the production of aqueous humor and increasing uveoscleral outflow. be. In Japan, 0.1% Aifagan ophthalmic solution containing 0.1% brimonidine tartrate was launched in 2012. Kotobuki Pharmaceutical obtained approval. Currently, it is widely used as a combination therapy for patients who are not sufficiently effective with first-line drugs such as prostaglandin-related drugs and ÿ-blockers alone.

Previous studies have reported neuroprotective effects of brimonidine. Optic nerve contusion using rats It has been confirmed that brimonidine suppresses secondary induced retinal ganglion cell (RGC) death through activation of ÿ2 receptors in a mouse model of normal-tension glaucoma6 ) . To In addition, brimonidine has been shown to suppress IOP-independent progression of RGC death 7) . Furthermore, in a tumor necrosis factor (TNF)-induced optic nerve degeneration model using rats, it was confirmed that brimonidine inhibited TNF-induced axonal degeneration in a dose-dependent. manner8 ).

In addition, in a clinical trial conducted in the United States in patients with normal tension glaucoma, 0.2% brimonidine tartrate eye drops were found to significantly suppress the progression of visual field damage compared to 0.5% timolol eye drops. 9) . Since there was no difference in the intraocular pressure-lowering effects of 0.2% brimonidine tartrate and 0.5% timolol eye drops, brimonidine has a direct neuroprotective effect apart from its intraocular pressure-lowering effects. It is thought that the protection of RGCs can also prevent the progression of visual field damage in glaucoma 9) . The 0.2% brimonidine tartrate ophthalmic solution used in the above study was The concentration of brimonidine in the vitreous when given was measured and confirmed to be higher than the concentration that activates the ÿ2 receptor. 10 )

In Japan, it has been confirmed that brimonidine in 0.1% Aifagan ophthalmic solution penetrates into the human vitreous. Possibility of drug therapy for retinal neuroprotection with Aifagan ophthalmic solution 0.1%11)12).

## 2.2 Significance of research

In September 2019, Senju Pharmaceutical obtained approval for ibeta combination ophthalmic solution containing 0.1% brimonidine tartrate and This eye drop contains 0.5% timolol (0.68% as timolol maleate). Timolol is a non-selective ÿ-blocker with a strong intraocular pressure-lowering effect. Because it suppresses aqueous humor production and has a strong effect of lowering intraocular pressure, it is widely used as a first-choice drug for the treatment of glaucoma along with prostaglandin-related drugs. By combining two active ingredients with different pharmacological actions, this drug has been confirmed to have an additive effect in lowering intraocular pressure.

Machine Translated by Google



It also contributes to the improvement of eye drops and avoids the weakening of the effect due to washout of the two eye drops when instilled without leaving an interval. It is expected to be a drug that provides a new option for the treatment of internal disorders.

Therefore, brimonidine was added to ibeta combination ophthalmic solution in the same way as ifagan ophthalmic solution 0.1%. In order to confirm whether retinal neuroprotection can be expected, we will examine the transfer of brimonidine to the vitreous body when ibeta combination ophthalmic solution is administered to humans .

Furthermore, in this study, we will confirm the correlation between ocular tissue drug concentration data and patient background, and investigate the factors that affect drug migration to the posterior segment of the human eye (retina, vitreous body, etc.). We believe that elucidation of these findings will be useful for the optimization of ophthalmic treatment and the development of eye drops for not only glaucoma but also posterior segment diseases.

# 3 Purpose and evaluation items

In order to investigate the possibility of drug therapy for retinal neuroprotection of ibeta ophthalmic solution, eye drops were administered to humans. To evaluate the migration of brimonidine into the vitreous body when administering beta combination ophthalmic solution.

# 4 Study design

## 4.1 Study design

Single-center, unshielded, uncontrolled study

## 4.2 Randomization/blinding

As this study is an uncontrolled study and has a single arm, there is no randomization or blinding.

# 5 Target population

## 5.1 Eligibility Criteria

### 5.1.1 Selection criteria

1) Subjects who can obtain voluntary written consent from the subject after receiving a sufficient explanation for participation 2) Age at the time of obtaining consent 20 years or older, regardless of gender 3) Surgery for premacular membrane or macular hole scheduled patient

Machine Translated by Google



### 5.1.2 Exclusion Criteria

1) Subjects with ocular

inflammation 2) Subjects with vitreous

hemorrhage 3) Subjects with proliferative

diabetic retinopathy 4) Subjects with severe corneal

epithelial disorder 5) Subjects with contraindications for ibeta combination ophthalmic solution or careful administration , and the principal investigator, etc.

Those who have determined that participation in the study is inappropriate

6) Subjects with a history of serious adverse reactions to ÿ2 agonists and ÿ- blockers

### 5.1.3 Rationale for setting

ÿ Selection criteria

1): Set for ethical considerations. 2): It was set to unify the

subject's background. 3): The target patients for this study were set.

•Exclusion criteria

This was set because it affects the subject's safety considerations and the primary evaluation of this study.

## 5.2 Target sample size

### 5.2.1 Target sample size

Target sample size: 10 cases

### 5.2.2 Basis for setting the number of cases

When Iphagan ophthalmic solution 0.1% was administered to humans, the vitreous brimonidine concentration per group was

At least 5 cases were evaluable12 ). In addition, in phase I trials for ophthalmic formulation development, the test drug group is usually 6

The number of cases in which safety and pharmacokinetics (plasma drug concentration) can be evaluated is set.

there is Therefore, in this study, the number of cases was set at 10, taking into account the drop-outs.

# 6 Intervention

## 6.1 Drugs and medical devices to be used

Machine Translated by Google



### 6.1.1 Overview of pharmaceuticals and medical devices

In this study, the following test drugs will be used.

test drug

| | |
|---|---|
| common name | Brimonidine tartrate/timolol maleate |
| Product name/Manufacturer/Marketer | Ibeta combination ophthalmic solution/Senju Pharmaceutical Co., Ltd. |
| Dosage form/property/content | Clear, pale greenish-yellow to greenish-yellow, sterile aqueous eye drops containing 1.0 mg of brimonidine tartrate and 5.0 mg of timolol (6.8 mg as timolol maleate) per 1 mL of eye drops |
| Therapeutic classification | Other ophthalmic agents |
| Efficacy/effect | If other glaucoma drugs are not effective for the following diseases: glaucoma, high ocular hypertension |
| Dosage and administration | 1 drop at a time, 2 times a day |
| storage conditions | Room temperature storage |

### 6.1.2 Anticipated side effects

The following side effects have been reported with the test drug 13). 1) Clinically significant adverse reactions (1) Ocular pemphigoid (incidence unknown) (2) Bronchospasm, dyspnea, respiratory failure (incidence unknown) (3) Heart block, congestive heart failure, cardiac arrest (incidence unknown) ) (4) Cerebral ischemia, cerebrovascular accident (incidence unknown) (5) Systemic lupus erythematosus (incidence unknown)

2) Other side effects

| | 1% to less than 5% | 0.1% to less than 1% | frequency unknown |
|---|---|---|---|
| hypersensitivity | | | Contact dermatitis, papules, rashes, erythema, hives |
| eye | Punctate keratitis, conjunctivitis Membrane congestion, eye irritation, ocular discomfort, corneal erosion | allergic Meningitis, ocular pruritus, photophobia, conjunctival edema, scintillating scotoma | eyelid erythema, eyelid edema, eyelid disorders, ptosis, pruritus, blepharitis, allergic blepharitis, meibomian gland infarction, conjunctivitis, conjunctival follicles, conjunctival pallor, conjunctival hemorrhage, dry keratoconjunctiva inflammation, keratitis, stye, iritis, cataract, vitreous detachment, vitreous floaters, abnormal sensation in the eye, foreign body sensation in the eye, eye |

Machine Translated by Google



| | | | |
|---|---|---|---|
| | | | Grease, eye pain, burning sensation, blurred vision, dry eye, asthenopia, increased tearing, visual disturbance, visual field defect, decreased vision, miosis, Corneal hyposensitivity, diplopia, edema/opacity of the macular fundus, corneal opacity |
| Cardiology | | | Arrhythmia such as hypotension, hypertension, palpitations, tachycardia, bradycardia, syncope, edema, Raynaud's phenomenon, cold extremities |
| respiratory | | | Nasal irritation, dry nose, rhinitis, sinusitis, cough, dyspnea, bronchitis, Pharyngitis |
| psychoneurotic system | | | Dizziness, vertigo, ear Crying, headache, paresthesia, fainting, evil Dreams, depression, depression, somnolence, insomnia, exacerbation of myasthenia gravis |
| Digestive organ | | | Dysgeusia, dry mouth, dry mouth, nausea, gastrointestinal disorders, indigestion, abdominal pain, diarrhea |
| Infection | | | Influenza syndrome, common cold, callus spiratory infection |
| others | | Pruritus Ear Warts, Anemia, Asthenia, Fatigue, Mood | Discomfort, discomfort, malaise, weakness, muscle Muscle pain, chest tightness, increased blood bilirubin, increased blood glucose, increased blood triglycerides, increased blood uric acid In addition, hypercholesterolemia |

## 6.2 Protocol treatment

### 6.2.1 Dosage and Administration

One drop of ibeta combination ophthalmic solution was applied to the eye to be evaluated twice a day for 8 days (7 days before surgery and on the day of surgery). morning and 2 hours before surgery (± 1 hour)).

In addition, when using other eye drops together, leave an interval of at least 5 minutes between the drops.

[Setting basis]

Machine Translated by Google



Follow the approved dosage and administration of ibeta combination eye drops. The grounds for setting the administration period, etc. are as follows. It is as follows.

1) Human vitreous penetration studies of brimonidine tartrate ophthalmic solution conducted in the United States and Japan10 )12 ) showed that retinal neuroprotective concentrations were transferred within about 7 days of administration. Also In view of the fact that the last outpatient day before hospitalization for patients scheduled for surgery in this department is about 7 days before surgery, and that preoperative treatment drugs (antibacterial agents, etc.) are usually prescribed and self-instillation is started at that time, the eye drop period is set to 8 days. days (approximately 7 days before surgery and on the day of surgery).

2) Regarding the administration time before surgery, in a human vitreous transfer study of brimonidine tartrate eye drops conducted in the United States10 ) , brimonidine concentration in the vitreous reached a peak 1 to 2 hours after administration. and the time to start preoperative preparation for vitrectomy surgery. It was decided to instill the eye drops 2 hours before.

3) In a phase I study of ibeta combination ophthalmic solution in healthy adults, The safety of eye administration has been confirmed. In addition, primary open-angle glaucoma (broad definition) and high vision In a phase III trial for hypertension, safety was confirmed at 52 weeks.

### 6.2.2 Packaging and Labeling

As the test drug, commercially available ibeta combination ophthalmic solution is used. Distinguish from drugs prescribed in ordinary medical practice The eye drop bag, etc. should be labeled as being for clinical research.

## 6.3 Management method

### 6.3.1 Storage and delivery

The study drug should be stored at room temperature. The principal investigator or study drug manager (if the study drug is managed by the pharmacy department, etc.) shall Create and maintain records regarding the status and returns of unused items.

### 6.3.2 Disposal/return

The principal investigator will retrieve all study medication from the subject after the study is completed, whether or not it has been opened. na The principal investigator or study drug manager shall return all unopened study drugs to Senju Pharmaceutical Co., Ltd., the study drug provider, after the study is completed or discontinued. Discard the opened study drug.

## 6.4 Combination therapy

### 6.4.1 Concomitant treatment

Machine Translated by Google



Follow the prescription for vitrectomy.

### 6.4.2 Concomitant therapy

There are no contraindicated drugs or therapies in this study.

### 6.4.3 Restricted concomitant therapy

There are no drugs or therapies with concomitant restrictions in this study.

### 6.4.4 Precautions for concomitant use

Since the following drugs interact with the study drug, caution should be exercised when using them concomitantly.

| Drug name, etc. | Clinical symptoms/measures | Mechanism/risk factor |
|---|---|---|
| Antihypertensive | May potentiate antihypertensive effect be. | Additively enhances antihypertensive effect It is thought that |
| central nervous system depressant • Barbituric acid derivatives • Opioid analgesics • Sedatives • Anesthesia, etc. alcohol | May enhance sedation be. | Additively enhances sedation It is thought that |
| Monoamine oxidase inhibitor | May affect blood pressure fluctuations be. | noradrenaline metabolism and thought to affect reuptake be done. |
| Concomitant use with omidenepag isopropyl | timolol maleate increased the incidence of ocular inflammatory side effects such as conjunctival hyperemia. Admitted. | mechanism unknown |
| adrenaline dipivefrin hydrochloride | It is reported that the mydriatic effect was promoted. I have a warning. | mechanism unknown |
| Catecholamine depletor: reserpine etc. | Excessive suppression of the sympathetic nervous system may lead to hypotension hypertension, bradycardia, dizziness, fainting, and orthostatic hypotension. | cause depletion of catecholamines Rubbing agents additively potentiate ÿ-blocking activity. |
| ÿ-blockers (systemic administration): Atenolol | Intraocular pressure lowering or systemic effects of ÿ-blockers may be enhanced. | The effect appears additively. |

Machine Translated by Google



| | | |
|---|---|---|
| Propranolol hydrochloride<br>Metoprolol tartrate | There is. | |
| Calcium antagonist:<br>verapamil hydrochloride<br>diltiazem hydrochloride | Atrioventricular conduction disturbance, left ventricular failure, and hypotension may occur. | Reciprocal action is enhanced. |
| Digitalis preparations:<br>Digoxin<br>digitoxin | If heart stimulation conduction disorder (bradycardia, atrioventricular block, etc.) occurs There it is. | Additively enhances action (inhibition of cardiac stimulation conduction). |
| Drugs with CYP2D6 inhibitory activity<br>Agent Quinidine sulfate hydrate<br>Selective serotonin reuptake inhibitor | It has been reported to enhance ÿ-blocking effects (e.g., decreased heart rate, bradycardia). | These drugs are timolol P450, a metabolic enzyme (CYP2D6) and thymo Roll blood concentration increases be. |

## 7 Research methods and procedures

### 7.1 Subject Recruitment

Outpatient visits to patients who may meet the eligibility criteria among those who are visiting medical institutions In some cases, the principal investigator, etc. will provide guidance regarding the research.

### 7.2 Obtaining Consent

Before the subject participates in the research, the principal investigator, etc. will hand over the explanatory document and the consent form to the subject, give them a full explanation, and obtain their voluntary consent. In the informed consent form, the principal investigator, etc. who provided the explanation and the subject Sign and date each. After obtaining consent, the principal investigator, etc., will provide the subject with a copy of the consent document with the signature and date. If the test was performed before consent was obtained, the results can be used after obtaining the subject's consent.

### 7.3 Subject Enrollment

Subject registration is performed using a subject identification code list. Enter the subject's background information such as date of birth and gender, and the subject's identification code in the subject identification code list. Since it is a single group and is not randomized, registration is by completing the subject identification code list.

complete the recording.

Machine Translated by Google



## 7.4 Observation items and information/procedures to be collected

During the study period, the subject visited the hospital according to the schedule shown in 1 Research Overview, and performed necessary observations and examinations. implement. The contents to be implemented and collected in observations and inspections are as follows.

1) Subject background factors

The principal investigator, etc. will investigate the following items and describe them in the case report form.

• Gender •

Date of birth (age) • Diagnosis

name

•Presence or absence of major eye complications (if yes, confirm the disease name) •Presence

or absence of major systemic complications (if yes, confirm the disease name) 2) Height/weight,

BMI

The principal investigator, etc., will measure the height (cm) and weight (kg), calculate the BMI using the formula below, and record it in the case report form.

BMI = Weight (kg) ÷ [Height (m) × Height (m)] 3) Tear volume

The principal investigator, etc., shall measure the amount of lacrimal fluid produced in the eye to be evaluated for 5 minutes in millimeters (mm) using Schirmer test strips, and record the results in the case report form. Measurements were taken under spontaneous eye blinks without the use of topical anesthetics. to be carried out.

4) Axial length

The principal investigator, etc., will measure the axial length of the eye to be evaluated using an ultrasonic eye/corneal pachymeter, and record the results in the case report form. If conducted within 120 days prior to Visit 1, it can be used as data for Visit 1. 5) Corneal thickness

The principal investigator, etc., will measure the corneal thickness of the eye to be evaluated using an ultrasonic eye axial length/corneal thickness measuring device, and record the results in the case report form. If conducted within 120 days prior to Visit 1, it can be used as data for Visit 1. 6) Corneal curvature radius

The principal investigator, etc., will measure the corneal curvature radius of the eye to be evaluated using an autorefractometer, and record the results in the case report form. If conducted within 120 days prior to Visit 1, it can be used as data for Visit 1. 7) Number of corneal endothelial cells

The principal investigator, etc., will measure the number of corneal endothelial cells in the subject eye using a specular microscope, and record the results in the case report form. If conducted within 120 days prior to Visit 1, it can be used as data for Visit 1.

8) best corrected visual acuity

Machine Translated by Google



The principal investigator, etc., will measure the distance visual acuity of the eye to be evaluated, and describe the value of the best corrected visual acuity in the case report form.

be. If the unaided visual acuity is 1.0 or higher, the unaided visual acuity can be the best corrected visual acuity. 9) Intraocular

pressure The principal investigator, etc., will measure the intraocular pressure (mmHg) of the eye to be evaluated using a non-contact

tonometer and record it in the case report form. 10) Ophthalmic findings The principal investigator, etc., shall observe the cornea, conjunctiva, lens, etc.

of the eye to be evaluated using a slit lamp microscope.

and recorded in the case report form.

• Observe corneal and conjunctival findings to confirm the presence or absence of corneal epithelial damage.

• Observe the phakic findings and determine whether the eye is phakic, aphakic or pseudophakic.

Confirm.

11) Blood pressure/pulse rate

The principal investigator, etc. will measure the systolic and diastolic blood pressure (mmHg) and pulse rate (beats/minute) after resting for at least 5

minutes, and record the results in the case report form. 12) Aqueous humor and vitreous collection

The principal investigator, etc. will collect 100 µL of aqueous humor and 500 µL of vitreous body during surgery, and record the start time of surgery in

the case report form. The time when the aqueous humor and vitreous are collected is regarded as the start time of the operation.

13) Confirmation of compliance with eye drops

The principal investigator, etc., should determine the subject's compliance with the study drug through an interview and an instillation diary.

Check whether you are instilling the prescribed number of times and the time of instillation.

It should be confirmed whether or not it has been carried out on a regular basis, and it should be recorded in the case report form. The instillation diary will be given to the subject

when the study drug is delivered, collected from the subject after the study drug administration is completed, and kept at the study institution. It will be carried out as much as

possible even at the time of discontinuation and dropout.

14) Confirmation of adverse events

Description of adverse events, timing of onset/disappearance, severity, treatment, outcome, assessment of severity, relationship to study drug

Gender etc. should be recorded in the chart and case report form. Conduct a follow-up survey if necessary.

## 7.5 Implementation Period and Registration Period

Implementation period: jRCT publication date to December 31, 2020

Registration period: jRCT publication date to September 25, 2020

Observation period: jRCT publication date to September 30, 2020

## 7.6 End of Registration Period and Observation Period

Machine Translated by Google



The enrollment period will end when the number of cases enrolled in this study reaches the target number of cases. However, the goal Subjects who have already consented as study candidates at the time the number of cases is reached will be It shall be possible to register even after the number of cases has been reached. In principle, subjects who have not obtained consent by the time the target number of cases is reached cannot be enrolled.

After the end of the enrollment period, the observation period will end when all subjects complete the prescribed visits.

# 8 How to obtain consent

## 8.1 Informed Consent

Informed consent is obtained from subjects prior to study participation. The principal investigator, etc., shall use a written informed consent document prepared in accordance with the Clinical Research Act and approved by an accredited clinical research review committee, and explain the research content in terms that are easy for the subject to understand. Allow sufficient time to answer subject questions and consider research participation. In addition, consent is based on the free will of the subject, and even if you do not agree, it will be disadvantageous. Emphasize that you will not be treated unfavorably, and that you can withdraw your consent at any time if you change your mind after giving consent, and that even in that case you will not be treated unfavorably. When obtaining consent to participate in the research, the signature of the doctor who provided the explanation and the subject, the date of explanation, and the date of consent should be written on the consent form. Explanatory Documents and Consent for Subjects Physician's consent form is kept at the medical institution. Any procedure related to the study Even if there is, it will be conducted after obtaining the subject's consent.

If new information that might affect the intention of the research subject was obtained during the study, which was not assumed at the time of consent acquisition, the informed consent document was promptly revised and explained to the research subject. Participate in research again confirm their intention to participate and obtain their consent. In addition, if there is a change in the research content, etc., consent will be obtained again in the same way. get.

## 8.2 Withdrawal of Consent

Even after consenting to participate in the study, the subject can withdraw consent at any time if they wish. withdrawal of consent When revising, the principal investigator, etc. will confirm the reason for withdrawal of consent and obtain a consent withdrawal document as much as possible. Study drug is discontinued when consent is withdrawn. Observations and tests at the time of discontinuation will be performed to confirm safety until the time of discontinuation of the study drug.

# 9 Adverse events and diseases, etc.

## 9.1 Definitions

Machine Translated by Google



### 9.1.1 Definition of adverse events

An adverse event is any unfavorable symptom, sign, disease, or laboratory abnormalities occurring in a subject. It does not matter whether or not there is a causal relationship with the research and the pharmaceuticals used in the research. Adverse events that occur after the start of administration are considered as adverse events. However, spontaneous exacerbation of symptoms of the underlying disease is not an adverse event. Make it not exist.

### 9.1.2 Definition of serious adverse events

If the investigator, etc. determines that the adverse event corresponds to the following criteria, it will be regarded as a serious adverse event.

1 Death or risk of death

2 Those who require hospitalization at a medical institution or an extension of the period of hospitalization for treatment

3 Disability or likely to lead to disability 4 Serious

according to 1 to 3 5 Congenital disease or

abnormality in later generations

Hospitalization for the reasons listed below is not considered a serious adverse event.

1) Elective treatment for pre-existing conditions unrelated to the target disease of this study or planned prior to study participation

2) Hospitalization for social reasons and caregiver's temporary rest without deterioration of general condition.

### 9.1.3 Definition of diseases, etc.

Among adverse events, in addition to disease, disability, death, or infection, abnormal laboratory test values and various symptoms suspected to be caused by the implementation of clinical research are defined as “disease, etc.” In addition, those caused by the implementation of clinical research are those that have a causal relationship with the pharmaceuticals used in the research or a causal relationship with the research procedure. shall be A causal relationship will be determined by the principal investigator, etc. based on 9.2 Causal Relationship with Research.

## 9.2 Research causality

The causal relationship should be determined not only by the time relationship with the start of interventional treatment, but also by the course of the underlying disease, complications, and concomitant disease. Make judgments by taking into account factors such as drugs, research procedures, accidents, and other external factors. The causal relationship is Judge and record according to the criteria below.

Causal **–** no matter known or unknown to be caused by the clinical study or intervention,

Judge according to the following.

**ÿ** Reasonably or reasonably likely to be attributed to the study or intervention

**ÿThere** is a temporal relationship with the research

**ÿNo** other cause can be shown, and a causal relationship with the research cannot be denied

• **Not Causal** – Judging according to the following criteria.

Machine Translated by Google



ÿ Not reasonably attributable to research or intervention

ÿ **Unable** to show temporal relationships

ÿ Other causes can be shown

## 9.3 Predictability

The predictability of adverse events will be determined based on the research protocol, package insert, and interview form. Yes If the nature, severity or frequency of the adverse event is inconsistent, it is considered an unknown adverse event.

## 9.4 Collection period and follow-up period

Adverse events, whether serious or non-serious, occurred from the first day of administration to the end of the observation period. Collect and record at Clinical findings present on the first day of administration should be considered as underlying diseases and not as adverse events. However, exacerbation of underlying disease will be recorded as an adverse event.

Non-serious adverse events determined to be causally related to study drug will be followed up until possible recovery or remission and all serious adverse events are followed until resolution or remission. However, death, disability, sequelae, etc. Follow-up will be terminated when the principal investigator determines that further recovery is difficult or follow-up is impossible. Non-serious adverse events unrelated to the study, regardless of outcome, should not be pursued specifically in the study. no.

## 9.5 Reporting Adverse Events

### 9.5.1 Reporting of all adverse events

All adverse events should be investigated and recorded as specified in 7.4 14) Confirmation of adverse events. Of all adverse events, serious adverse events should be reported according to 9.5.2 Reporting of serious adverse events, Diseases, etc. for which a causative relationship cannot be denied shall be reported based on 9.5.3 Reports of diseases, etc.

### 9.5.2 Reporting of serious adverse events

If a serious adverse event occurs, the research subinvestigator who knows the information should promptly report it to the principal investigator. be. The principal investigator shall promptly grasp the serious adverse events and the status of responses, and consider necessary responses. Regardless of whether there is a causal relationship, serious adverse events should be reported to Senju Pharmaceutical Co., Ltd. orally, by phone, by e-mail, etc., in the same way as other adverse events. If a causal relationship cannot be denied, a report shall be made based on 9.5.3 Reports of diseases, etc., in addition to the procedures in this section. In addition, follow the regulations and procedures of the implementing medical institution. necessary measures, such as reporting to the administrator of the medical institution.

### 9.5.3 Reporting illness, etc.

Machine Translated by Google



If any of the diseases, etc., that fall under any of the following 1 to 5 occur, the research co-investigator who knows

report to the attending physician. The principal investigator who receives the report reports to the manager of the medical institution. then

Report to the accredited clinical research review committee and the Minister of Health, Labor and Welfare by the following reporting deadlines, depending on the predictability of the disease, etc.

do.

1 Death or risk of death

2 Those who require hospitalization at a medical institution or an extension of the period of hospitalization for treatment

3 Disability or risk of disability 4 Serious according

to 1 to 3

5 Congenital diseases or abnormalities in subsequent generations

**Submission deadline for unapproved/off label use**

| | Type of adverse event | causality | predictability | Committee Reporting deadline | Minister of Health, Labor and Welfare Reporting deadline |
|---|---|---|---|---|---|
| ÿ | 1 death or may lead to death | can be | can not | 7 days | The 7th |
| | | | 15 days | | |
| | | none | | | |
| ÿ | 2 Hospitalization for treatment or extension of hospitalization period<br>3 Obstacles/fear of injury<br>4 death or serious according to 2-4<br>5 Congenital diseases or abnormalities in subsequent generations | can be | 15 days you can't | | 15th day |
| | | | can | | |
| | | none | | | |

When reporting to the accredited clinical research review board, use the uniform form 8 and prepare it by the principal investigator.

be. In addition, the principal investigator will report to the administrator of the medical institution and, if necessary, obtain the cooperation of the

administrator of the medical institution and take preventive measures and countermeasures against the occurrence of diseases, etc.

When reporting to the Minister of Health, Labor and Welfare, we will use the clinical research implementation plan and research summary publication system maintained by the Ministry of Health, Labor and Welfare.

Do it at Stem (jRCT: https://jrct.niph.go.jp/).

All diseases, etc. that occur in relation to clinical research are registered as certified clinical

Report the occurrence of diseases, etc. to the research review committee and the administrator of the medical institution.

# 10 Cancellation and Termination

## 10.1 Subject Withdrawal

If the investigator, etc. meets the following criteria during the research period, the research subject will be discontinued.

do.

• Stop criteria

1) Discontinuation of administration is deemed appropriate by the principal investigator, etc., due to worsening symptoms of the primary disease that make it impossible to continue the study.

if you judge

Machine Translated by Google



2) Due to the occurrence of adverse events that make it impossible to continue the study, or exacerbation of complications, the principal investigator etc.

If it is determined that cancellation is appropriate

3) When the subject dies 4) When the

subject requests withdrawal of consent after consent is obtained 5) When the surgery itself

is canceled deviation, conflict with exclusion criteria). However, if an active ophthalmologic

disease develops during the study period, there is no possibility that the principal investigator, etc. will affect the ophthalmologic examination,

or there is no problem with the subject's safety.

The research may be continued if it is judged to be inappropriate.

7) When the subject does not come to the hospital after obtaining consent 8)

When the subject becomes pregnant after obtaining consent

• Basis for setting

1) 2) 8) 9): Set for safety considerations for subjects. 3) 5) 7): Set because the

research cannot be continued.

4): Set for ethical considerations for subjects. 6): For subjects who should

not be administered, it was set because it is desirable to discontinue the study early.

If a subject is found to meet the discontinuation criteria, the principal investigator, etc., will explain to that effect and discontinue administration of the study drug. At the time of discontinuation, observations and examinations at the time of discontinuation and withdrawal should be performed promptly (on the same day as possible) after judging that the study should be discontinued, and the date of discontinuation (the day when the discontinuation was judged), the reason for discontinuation, etc. should be recorded in the case report form. na In addition, if it is found that the subject cannot come to the hospital due to circumstances, the principal investigator, etc. confirm.

## 10.2 Termination of study

If the following situation occurs, the principal investigator, the accredited clinical research review board, or the administrator of the medical institution If it is determined that it should be discontinued, the entire study may be discontinued.

•When an unpredictable serious illness occurs and there is concern that it may be detrimental to the entire subject.

•When serious violation/non-compliance with the law, related laws and regulations or research protocol is found

•When facts that impair or may impair ethical validity or scientific rationality •When a serious risk to the subject is identified •When an accredited clinical research review board gives an opinion

•When the Minister of Health, Labor and Welfare issues a request or recommendation to stop

Machine Translated by Google



In case of discontinuation, the investigator will report to the accredited clinical research review board and the administrator of the medical institution. further , contact the subject and inform them of any changes to the study schedule. In the event of discontinuation, the subject shall be instructed to immediately discontinue administration of the study drug, and observations and examinations at the time of discontinuation and withdrawal shall be performed.

## 10.3 End of research

The research will end when all of the following items are completed.

•Completion of enrollment of subjects in the study and completion of the observation period •Creation of the primary endpoint report, review report, and summary of the review report

•Submission of the primary endpoint report to the Minister of Health, Labor and Welfare

•Submission of summary report and research plan to Minister of Health, Labor and Welfare

•Submission of the primary endpoint report, review report, and summary of the review report to the administrator of the implementing medical institution

•Submission of the primary endpoint report and review report to the funders Registration of summary •Report to the administrator of the medical institution that the research results have been published

# 11 How to minimize expected benefits/disadvantages and risks

## 11.1 Expected Benefits

There is currently no direct benefit to subjects in this study. However, glaucoma, which is a representative disease that presents retinal visual dysfunction, is the leading cause of blindness in Japan. New for patients with glaucoma

We believe that this will lead to the provision of effective treatment methods. In the future, it will also be used as a neuroprotective treatment for diabetic retinopathy, conservative therapy up to repositioning after retinal detachment, retinitis pigmentosa, etc., and protection against retinal neuropathy due to surgical invasion during retinal and vitreous surgery. It has therapeutic potential.

## 11.2 Anticipated Disadvantages

The investigational drug has already obtained marketing approval for glaucoma, and although there are no notable disadvantages, there are adverse events associated with brimonidine and timolol. Adverse events reported in Japan to date are reversible and will resolve after discontinuation of administration.

## 11.3 How to minimize risk

Machine Translated by Google



In the event of illness, etc. caused by the test drug administered in this study, including suspension of treatment or discontinuation of study participation Consider the response individually. In addition, blood pressure and pulse were measured before and after administration to confirm the systemic effects of eye drops. admit. To confirm the effects of eye drops on the anterior segment of the eye, corneal and conjunctival findings should be checked before and after administration.

# 12 Ethical Matters and Considerations

## 12.1 Compliance with Laws and Guidelines

As this research will be funded by a pharmaceutical company, it falls under specified clinical research under the Clinical Research Law (Law No. 16 of 2017). Therefore, this study complies with the Clinical Research Act and the Declaration of Helsinki Conduct in accordance with ethical principles based on

Legally required materials, including research protocols and protocols, are subject to review and approval by an accredited clinical research review board. After that, with the permission of the administrator of the implementing medical institution, the study will start after completing registration with jRCT. If there are any changes to these materials, etc., the same procedures will be followed before the changes are implemented.

## 12.2 Accredited Clinical Research Review Board

This research will receive review opinions from the following accredited clinical research review committee. in unavoidable circumstances Except for this, in principle, the committee that receives review opinions will not change throughout the entire research period.

Name: National University Corporation Fukui University Clinical Research Review

Committee Accreditation number: CRB5180014

Location: 23-3 Shimoaitsuki Matsuoka, Eiheiji-cho, Yoshida-gun, Fukui Prefecture

Contact: 0776-61-8640

E-mail address: rinrisys@ml.u-fukui.ac.jp

## 12.3 Handling of personal information, etc.

### 12.3.1 About anonymization

Data including personal information of subjects collected in this study will be Delete the information and give the subject identification code for research. The subject identification code is the chart ID and the rule A combination of non-gendered alphanumeric characters, common code AV and case number alphanumeric characters (A1V1, A2V2, …). Also, create and store a subject identification code list for identifying individual subjects. Monitoring will be conducted by a person appointed by the principal investigator.

Machine Translated by Google



### 12.3.2 Subjects' Rights Regarding Personal Information

The principal investigator shall, at the request of the subject, etc., disclose personal information, notify the purpose of use, make corrections, additions, deletions, and suspend use of personal information and personal identification codes held in the course of research. However, if there is a risk of harming the rights of the conducting medical institution, the proper conduct of clinical research, or the rights of the subject,

If the request is not accepted for a valid reason, explain the reason to the subject.

A fee may be collected for the disclosure of personal information, and if the medical institution has established a fee in its regulations, etc., it will be followed. If there is no provision for fees, the principal investigator shall determine the reasonable amount of fees, contact point for requesting disclosure, documents related to the request, method of identity verification, and method of collecting fees.

be.

## 12.4 Compensation for health damage

If a subject suffers a health hazard, appropriate treatment will be provided within the scope of insurance medical treatment. Self-pay portion of medical expenses Senju Pharmaceutical Co., Ltd. will pay. In this study, because the use of approved drugs is not covered by insurance,

To provide coverage, enroll in clinical research insurance. In the event of health damage for which a causal relationship with this research cannot be denied, compensation will be provided according to the degree of health damage and the content of the clinical research insurance contract. However, if the subject is negligent, it will not be covered. In addition, if the doctor is at fault, compensation will be provided under the doctor's liability insurance.

cormorant. Clinical research insurance is contracted promptly after initial approval is obtained.

## 12.5 Financial Burden or Honorarium for Subjects

The ibeta combination ophthalmic solution used in this study is provided free of charge by Senju Pharmaceutical Co., Ltd.

No financial burden is incurred. In addition, expenses related to other treatments, medical examinations, examinations, etc. performed within the scope of insurance medical treatment

The use of the subject will be paid for by the subject's health insurance and the subject's own expense. Since the frequency of hospital visits does not increase significantly compared to regular medical care, the economic burden on subjects due to participation in research is less than that of regular medical care.

no different. No rewards or other payments will be made to the subjects for their participation in the research.

# 13 Research Funds, Conflicts of Interest and Disclosure of Information

## 13.1 Research funding sources

This research is funded by Senju Pharmaceutical Co., Ltd. as a contract research. ibe used in research

The data combination ophthalmic solution was provided free of charge by Senju Pharmaceutical Co., Ltd. In addition, audit work, data management work and statistical analysis work will be performed by Senju Pharmaceutical Co., Ltd. free of charge. Conclude a consignment contract when receiving funds, drugs and services.

Machine Translated by Google



## 13.2 Conflict of interest situations

In this study, a consignment contract was signed between Senju Pharmaceutical Co., Ltd. and the University of Fukui, and Dai Inatani, the principal investigator, Receive research funds, drugs and services. Conflicts of interest arising in this research as a whole and conflicts of interest of individual physicians must be reported and confirmed before the start of the research.

Conflict of interest management plans will be formulated based on the results of fact-checking of conflicts of interest, and after obtaining approval from the accredited clinical research review committee, appropriate management and disclosure will be conducted in accordance with the review results. Conflicts of interest will also be disclosed when research results are published. Conflicts of interest will also be disclosed to research subjects.

Appropriate re-reporting and obtaining approval even after the start of research. For example, if there is a change in the conflict of interest status, the research responsibility When adding or changing physicians/sub-investigators, when periodic reports are made, or at other appropriate timings, review the conflict of interest status/conflict of interest management plan, etc. Get approval.

Personal conflicts of interest that should be disclosed in this research are as follows. Chief investigator (Inatani Univ.) receives over ¥1 million in personal benefits annually from Senju Pharmaceutical Co., Ltd.

## 13.3 Methods of Information Disclosure

The research summary and research results of this research will be published in the clinical research implementation plan and research summary publication system maintained by the Ministry of Health, Labor and Welfare. system (jRCT: https://jrct.niph.go.jp/) and submit an implementation plan to the Minister of Health, Labor and Welfare to publish it in jRCT. If there are any changes to the content, the jRCT should be revised, the approval of the accredited clinical research review board should be obtained, and the changes should be reported to the Minister of Health, Labor and Welfare. We also update the information at least once a year as required by law.

## 13.4 Publication of results

The results of this study will be made public by submitting to ``J Ocul Pharmacol Ther'' and other publications, and making presentations at ``World Glaucoma Congress'', ``Japanese Society of Clinical Ophthalmology'', and other means. In addition, the results of the primary endpoint and the total A summary report will be submitted to the Minister of Health, Labor and Welfare around September 2021 within one year after the end of all observation periods. rice field However, regarding the publication of the jRCT, we aim to publish it within 2021 after the paper is published. Publication of the paper is scheduled for 2021.

# 14 Statistical Matters

## 14.1 Analysis population

Machine Translated by Google



All cases collected after the end of the study were examined, and each analysis target population shown below was selected.

decide.

[Population for pharmacokinetic analysis]

All subjects enrolled in the study met the inclusion criteria and did not meet any of the exclusion criteria.

Instead, it should be a cohort of subjects who received the prescribed dose of study drug.

[Safety analysis population]

Of all subjects enrolled in the study, subjects who never received study drug, first visit

Subjects whose safety could not be evaluated due to reasons such as no revisit after that time will be excluded. The ophthalmologic test data for safety evaluation shall be applied to the eyes to be evaluated.

## 14.2 Statistical analysis

### 14.2.1 Analysis of the primary endpoint

[Pharmacokinetic

analysis] 1) Brimonidine concentration in aqueous humor and vitreous

Aqueous humor and vitreous brimonidine concentrations were measured in the pharmacokinetic analysis population, tabulated and analyzed.

conduct.

### 14.2.2 Analysis of secondary endpoints

[Pharmacokinetic

analysis] 1) Timolol concentration in aqueous humor and vitreous

Aqueous humor and vitreous timolol concentrations were measured in the pharmacokinetic analysis population, tabulated and analyzed.

cormorant. 2) Correlation between drug concentration data (aqueous and vitreous brimonidine and timolol concentrations) and patient background

Moroll concentration) was used as the objective variable, and explanatory variables were background factors (sex, age), height, weight, BMI, tear volume, axial length, corneal thickness, corneal curvature radius, corneal endothelial cell count, and ophthalmologic findings (corneal•Conjunctival findings, post-administration) It is examined by multiple regression analysis with

[Safety assessment]

1) Adverse events

Calculate the incidence of observed adverse events and adverse drug reactions (cases with occurrence/cases accepted for safety).

2) Visual acuity, intraocular pressure, ophthalmologic findings (cornea, conjunctiva), blood pressure, pulse Each item will be compared before and after administration.

Machine Translated by Google



### 14.3 Modifying the Statistical Analysis Plan

If the plan described in the research protocol is to be changed, the reason for the change and the impact of the change on the research results and overall

The investigator will carefully consider the impact, ethical and scientific validity of the impact. Any major changes to the primary endpoint and its analysis

should be reviewed and deemed justified.

and an outline of the changes will be specified in the research plan and revised.

The revised research protocol will be reviewed by an accredited clinical research review board, and changes will be made after hearing opinions.

Details of the changes will also be explained in the summary report.

## 15 Preparation of case report forms

The principal investigator, etc., will accurately prepare a case report form based on the source documents and submit a copy of it to the person in charge of data management. If there are any corrections to the information on the case report, make sure that the records before corrections are visible.

correct.

## 16 Storage and Disposal of Specimens and Information

### 16.1 Storage method and storage period

#### 16.1.1 Sample storage method and storage period

Aqueous humor and vitreous collected from subjects in this study will be attached with a subject identification code and stored frozen in a lockable deep freezer in Ophthalmological Laboratory 1. They are then shipped to drug concentration measurement institutions. Subject consents

Even if the request is withdrawn, the same measures will be taken for the samples obtained up to that point.

#### 16.1.2 Storage method and storage period of information

Paper-based information other than charts will be stored by the principal investigator in a lockable locker at the medical institution during and after the study period. The information to be stored shall be as follows, and the storage period shall be 5 years after the end of the research.

do. In addition, medical charts will be stored according to the regulations of the medical institution.

• Research plan, implementation plan •

Explanatory document and consent document for research subjects •

Primary endpoint report, summary report and its summary

• Notification of review results received from the accredited clinical research review committee

• Copies of reports to the Minister of Health, Labor and Welfare other than the implementation

plan • Source materials

Machine Translated by Google



• documentation on monitoring;

• Contract for implementation of specified clinical research

• Records related to the management of

pharmaceuticals, etc. • Other important documents related to research designated by the investigator

### 16.2 Disposal method

#### 16.2.1 Sample disposal method

At the end of the study, discard after drug concentration measurements are completed. Deleted the subject identification code attached to the sample and dispose of it as infectious waste in accordance with the waste management regulations.

#### 16.2.2 How to dispose of information

After the research is completed, the information can be discarded after the storage period stipulated in the research protocol has passed. Paper-based materials shall be shredded or dissolved using a shredder such as a cross-cut with a small shred size to make it irreproducible before disposing of it. Even if the subject withdraws consent,

We will keep the information until the retention period expires.

### 16.3 Safety management method

Sufficient security control measures shall be taken when storing specimens and information. Samples are strictly managed in a lockable deep freezer in Ophthalmology Laboratory 1, where access is controlled. Paper-based information will be strictly managed in a lockable cabinet in the ophthalmology office where access is controlled. These research data can only be accessed by investigators, subinvestigators and collaborators of research organizations approved by an accredited clinical research review board.

In addition, the subject identification code list will not be provided to Senju Pharmaceutical Co., Ltd., which manages the data.

The person in charge performs data management work in a state where individuals cannot be identified.

## 17 Quality control and quality assurance

### 17.1 Source material

The source materials for this research are as follows. Medical record,

subject identification code list, informed consent form, ophthalmic diary, serious adverse event report, study drug management table

Machine Translated by Google



Institutions and principal investigators are responsible for monitoring, auditing, and accredited clinical research related to clinical research. Provide direct access to all clinical research-related records, including source documents, for review by review boards and regulatory authorities.

## 17.2 Data management

In this study, Senju Pharmaceutical Co., Ltd. performed data management, including quality checks. cormorant. For details, follow the separately defined data management plan and other procedures.

## 17.3 Monitoring

The study will be conducted by a monitoring person appointed by the principal investigator. For more information, monitor Specified in the ring procedure manual. The name of the person in charge of monitoring will be specified in a separate nomination form.

## 17.4 Audit

In this research, in order to confirm and guarantee compliance with the Clinical Research Act, related laws and regulations, and the research protocol, Conduct audits. Details are specified in the audit procedure manual.

# 18 Legal reporting and information sharing, etc.

## 18.1 Who to report to and scope of information sharing

Various reports, notifications, information sharing, etc. will be carried out as follows.

| | Review request to committee Request/Report/Notify | Report to Minister of Health, Labor and Welfare notification/submission | Management of medical institutions report/submission to |
|---|---|---|---|
| Implementation plan (new) | ● | jRCT Registration ● | permission application |
| Implementation plan to Minister of Health, Labor and Welfare Completion of drawing submission (completion of publication) | ● | | ● |
| Implementation plan (change) | ● | jRCT Modification •*1 | permission application |
| 9.5.3 Illness report | ● | ÿ*3 | ● |
| Nonconformity | •*4 | | ● |
| regular report | ● | ● | ● |
| Primary endpoint report*5 | ● | ● | ● |

Machine Translated by Google



| | | | |
|---|---|---|---|
| Publication of the primary endpoint report table complete | | | ● |
| Comprehensive report | ● | | ● |
| Overview of the comprehensive report | ● | ● | ● |
| Research plan, written informed consent Calligraphy (at the end of research) | | ● | ● |
| Canceled research | ● | ● | |
| Contents of opinions from the committee | | | ● |
| Complaints about research conduct Accusation*2 | | | ● |

*1 Submit the changed implementation plan and notification form (implementation plan item change notification form or implementation plan minor change notification form).

*2 If a major nonconformity is confirmed through complaints or accusations, the major nonconformity is reported.

*3 Only illnesses within the scope stipulated in “9.5.3 Illness Report” are reported to the Minister of Health, Labor and Welfare.

*4 If a particularly serious nonconformity occurs, promptly report it to the accredited clinical research review committee and hear its opinion.

*5 Not required if a summary report is submitted and a primary endpoint report is not prepared.

## 18.2 Creation/Change, etc. of Implementation Plan

For the new review before the start of the study, the investigator should submit the implementation plan to the accredited clinical research review board. issued and approved. After approval, obtain approval for conducting research from the administrator of the medical institution and then register with jRCT and submit an implementation plan to the Minister of Health, Labor and Welfare. If your submission has been accepted and published on jRCT, please indicate so. Notify the accredited clinical research review board and report to the administrator of the medical institution.

The same shall apply when changing the implementation plan, but the accredited clinical research review board and the Minister of Health, Labor and Welfare shall Submit a minor change notification form for the implementation plan or a change notification form for the implementation plan together with the implementation plan after the change be.

## 18.3 Periodic reporting

Starting from the date of submission of the implementation plan to the Minister of Health, Labor and Welfare, regular reports will be made every year. From the date of jRCT publication Within two months after each year, the principal investigator will submit the Standard Form 5 Periodic Report, Standard Form 6 Periodic Disease Prepare a report such as the attached form 3 regular report and submit it to the accredited clinical research review committee. approved by the committee If approved, submit Form 3 Periodical Report to the Minister of Health, Labor and Welfare within one month of approval.

In addition, the principal investigator should report to the administrator of the medical institution using the above-mentioned regular report, etc. cormorant.

## 18.4 Nonconformity reporting

Machine Translated by Google



If it becomes clear that the clinical research does not conform to this research protocol or laws and regulations, The sub-investigator who becomes aware of the suitability will report to the principal investigator. The principal investigator will promptly report to the administrator of the medical institution. The principal investigator will appropriately consider recurrence prevention measures. If the principal investigator determines that the nonconformity that has occurred is particularly serious, the principal investigator must promptly prepare a Standard Form 7 Serious Nonconformance Report, submit it to the accredited clinical research review board, and provide its opinion. listen.

Serious nonconformities are mainly defined as follows.•Things that affect the

human rights and safety of research subjects •Things that affect the

progress of research and the reliability of results

In addition, if there is a concern that the investigator may not report the non-compliance to the appropriate reporting party, Sub-investigators can report directly.

Of the non-compliances, those in which the research protocol was not followed for unavoidable medical reasons, such as to avoid immediate danger to research subjects, are not included in serious non-compliances. Therefore, it is unavoidable that In the event of conformity, there is no need to report to the accredited clinical research review board, but even in such cases, appropriate records of nonconformity shall be created.

## 18.5 Illness Report

Illness reports shall be submitted by the principal investigator to an accredited clinical research review committee and the welfare Report to the Minister of Labor. The principal investigator will report to the administrator of the medical institution that the above report has been made and the nature of the disease, etc.

## 18.6 Reports on study discontinuation

If the study is discontinued, the principal investigator will prepare a standardized form 11 Discontinuation Notice within 10 days from the date of discontinuation. and notify the accredited clinical research review board. Also, prepare Form No. 4 Designated Clinical Research Discontinuation Notification and submit it to the Minister of Health, Labor and Welfare. Even if the study is discontinued, the primary endpoint report, clinical study report, and summary thereof should be prepared appropriately.

etc. will be reported.

## 18.7 Report on completion of research and preparation of report

When the principal investigator prepares the primary endpoint report, the investigator hears the opinion of the accredited clinical research review board and approves it. After approval, promptly register with jRCT, submit to the Minister of Health, Labor and Welfare, and publish. In addition, the principal investigator will submit a primary endpoint report to the administrator of the medical institution.

When the principal investigator prepares the summary report and the outline of the summary report in Attached Form 1, he/she should also listen to the opinion of the accredited clinical research review committee, register the summary with jRCT within one month after approval, and submit it to the welfare office. Minister of Labor

Machine Translated by Google



, together with the research plan and informed consent document, and publish it. The investigator responsible for the study is the administrator of the medical institution In addition to submitting a summary report and its summary, we will make a public report. In addition, the final research plan attached.

If the summary report is published in the jRCT, the principal investigator should submit a uniform form 12 and attach Attached Form 1 to notify the accredited clinical research review committee of the completion of the study.

### 18.8 Miscellaneous

The principal investigator will report the contents of the opinion to the manager of the medical institution when the accredited clinical research review board provides an opinion. After that, the principal investigator will cancel, suspend, or terminate the study according to the stated opinion. Consider appropriate measures, including In case of discontinuation, 10.2 Discontinuation of Research and 18.7 End of Research and Report Follow the report on the creation of

If a complaint or accusation is received regarding the research, the person who received the complaint or accusation shall promptly report it to the principal investigator, who shall report it to the manager of the medical institution. Principal investigator confirms the content of the complaint/accusation and consider necessary measures.

## 19 Research system

### 19.1 Research organization

• Implementing medical institution and investigator

University of Fukui Hospital Ophthalmology Professor Dai Inatani

• Funding

Senju Pharmaceutical Co., Ltd.

•Person in charge of monitoring

Fukui University Hospital Department of Ophthalmology, Associate Professor Yoshihiro Takamura

• Person in charge of audit

Senju Pharmaceutical Co., Ltd. Pharmaceutical Affairs Department Clinical Audit Group / Group Manager Tokuji Inui

• Data management manager

Eriko Kunikane, Clinical Research Promotion Group, Drug Fostering Research Promotion Department, Research and Development Division, Senju Pharmaceutical Co., Ltd.

•Statistical analysis manager

Eriko Kunikane, Clinical Research Promotion Group, Drug Fostering Research Promotion Department, Research and Development Division, Senju Pharmaceutical Co., Ltd.

Machine Translated by Google



## 19.2 Contact point

Consultations and inquiries from subjects, etc. will be accepted at the consultation desk described in the explanatory document. Also, the entire study

Consultations and inquiries regarding the above are accepted at the following counters.

| | |
|---|---|
| Implementing medical institution name | Fukui University Hospital |
| location | 23-3 Shimoaitsuki Matsuoka, Eiheiji-cho, Yoshida-gun, Fukui Prefecture 910-1193 |
| Affiliation/Position | Ophthalmology/Professor |
| Name of person in charge | Dai Inatani |
| phone number | 0776-61-8403 |
| E-mail address | inatani@u-fukui.ac.jp |

## 19.3 Outsourcing

In this research, audit work, data management work, and statistical analysis work were performed by Senju Pharmaceutical Co., Ltd. (Hyogo Prefecture). 6-4-3 Minatojima Minami-cho, Chuo-ku, Kobe). In addition, drug concentration measurement services are provided by CMIC Pharma. The work will be outsourced to Science Co., Ltd. (10221 Kobuchizawa-cho, Kitasha City, Yamanashi Prefecture). The investigator responsible for the research is entrusted Receive regular reports on the implementation status of outsourced work based on the contract, and provide guidance as necessary. supervised by

# 20 Others

## 20.1 Revision history

| Edition number | Created date | Enforcement date | change point | Reason for change |
|---|---|---|---|---|
| Version 1.0 | 2019/12/25 | | | First edition created |
| Version 1.1 | March 2, 2020 | | •Addition of grounds for setting research subjects (1.1)<br>•Recovery and disposal of study drug after completion of research Added description about (6.3.2) | In response to the points pointed out by CRB change accordingly |
| | | | | |
| | | | | |
| | | | | |
| | | | | |
| | | | | |

Machine Translated by Google



# 21 References

1. Japanese Glaucoma Society. Glaucoma clinical practice guideline 4th edition. 2018 (http://www.nichigan.or.jp/member/guideline/glaucoma4.pdf)

2. Iwase A, Suzuki Y, Araie M, Yamamoto T, Abe H, Shirato S, et al. Tajimi Study Group, Japan Glaucoma Society. The prevalence of primary open-angle glaucoma in Japanese: the Tajimi Study. Ophthalmology. 2004;111:1641-8. Ministry of Health, Labor and Welfare Research Grant

Kim Research Project for Overcoming Intractable Diseases Research on retinal choroidal and optic nerve atrophy FY2005 Supervisor Research report 26. Causes and Transition of Visual Impairment-Comparison between the Current Survey and the 1988 Survey. 2006;99-103.

4. The Collaborative Normal-Tension Glaucoma Study group. pressure reduction in the treatment of normal-tension glaucoma. Am J Ophthalmol. 1998; 126: 498-505.

5. The Collaborative Normal-Tension Glaucoma Study group. progression between untreated patients with normal-tension glaucoma and patients with therapeutically reduced intraocular pressure. Am J Ophthalmol. 1998; 126: 487-97.

6. Yoles E, Wheeler LA, Schwartz M. ÿ2-adrenoreceptor agonists are neuroprotective in a rat model of optic nerve degeneration. Invest. Ophthalmol. Vis. Sci. 1999;40:65-73.

7. Semba K, Namekata K, Kimura A, Harada C, Mitamura Y, Harada T. Brimonidine prevents. neurodegeneration in a mouse model of normal tension glaucoma. Cell Death Dis. 2014;5:e1341.

8. Kitaoka Y, Kojima K, Munemasa Y, Sase K, Takagi H. Axonal protection by brimonidine with modulation of p62 expression in TNF-induced optic nerve degeneration. Graefes Arch Clin Exp Ophthalmol. 2015;253:1291-6.

9. Krupin T, Liebmann JM, Greenfield DS, Ritch R, Gardiner S; Low-Pressure Glaucoma Study Group. A randomized trial of brimonidine versus timolol in preserving visual function: results from the low-pressure glaucoma treatment study. Am J Ophthalmol. 2011;151:671-81.

10. Kent, AR, Nussdorf, JD, David, R., Tyson, F., Small, D., Fellows, D. Vitreous concentration of Topically applied brimonidine tartrate 0.2%. Ophthalmology. 2001;108:784-7.

11. Shinno K, Kurokawa K, Kozai S, Kawamura A, Inada K, Tokushige H. The relationship of brimonidine concentration in vitreous body to the free concentration in retina/choroid following topical administration in pigmented rabbits, Curr. Eye Res. 2017;42:748-53.

12. Takamura Y, Tomomatsu T, Matsumura T, Takihara Y, Kozai S, Arimura S, et al. and aqueous concentrations of brimonidine following topical application of brimonidine

Machine Translated by Google



tartrate 0.1% ophthalmic solution in humans. J Ocul Pharmacol Ther. 2015;31:282-5.
